# Isolation and characterization of a T7-like lytic phage for *Pseudomonas fluorescens*

**DOI:** 10.1186/1472-6750-8-80

**Published:** 2008-10-27

**Authors:** Sanna Sillankorva, Peter Neubauer, Joana Azeredo

**Affiliations:** 1 IBB-Institute for Biotechnology and Bioengineering, Centre of Biological, Engineering, Universidade do Minho, Campus de Gualtar 4710-057, Braga, Portugal; 2 Bioprocess Engineering Laboratory, Department of Process and Environmental Engineering and Biocenter Oulu, University of Oulu, P.O. Box 4300, FI-90014 Oulu, Finland

## Abstract

**Background:**

Despite the proven relevance of *Pseudomonas fluorescens *as a spoilage microorganism in milk, fresh meats and refrigerated food products and the recognized potential of bacteriophages as sanitation agents, so far no phages specific for *P. fluorescens *isolates from dairy industry have been closely characterized in view of their lytic efficiency. Here we describe the isolation and characterization of a lytic phage capable to infect a variety of *P. fluorescens *strains isolated from Portuguese and United States dairy industries.

**Results:**

Several phages were isolated which showed a different host spectrum and efficiency of lysis. One of the phages, phage ϕIBB-PF7A, was studied in detail due to its efficient lysis of a wide spectrum of *P. fluorescens *strains and ribotypes. Phage ϕIBB-PF7A with a head diameter of about 63 nm and a tail size of about 13 × 8 nm belongs morphologically to the *Podoviridae *family and resembles a typical T7-like phage, as analyzed by transmission electron microscopy (TEM). The phage growth cycle with a detected latent period of 15 min, an eclipse period of 10 min, a burst size of 153 plaque forming units per infected cell, its genome size of approximately 42 kbp, and the size and N-terminal sequence of one of the protein bands, which gave similarity to the major capsid protein 10A, are consistent with this classification.

**Conclusion:**

The isolated T7-like phage, phage ϕIBB-PF7A, is fast and efficient in lysing different *P. fluorescens *strains and may be a good candidate to be used as a sanitation agent to control the prevalence of spoilage causing *P. fluorescens *strains in dairy and food related environments.

## Background

*Pseudomonas fluorescens *is a Gram-negative psychrotrophic bacterium that can be divided into five biovars (I through V). This bacterium has frequently been isolated from milk and food related environments and characterized due to its relevant spoilage activity. *Pseudomonas *spp. are the main concern with regard to proteolytic degradation of milk, along with other less proteolytic degradative and milk coagulant organisms such as *Achromobacter*, *Aeromonas*, *Flavobacterium*, and *Xanthomonas *spp. [1-5].

Studies with phages as control organisms of bacterial infections have increased in the recent years mainly due to the emergence of bacterial resistance to a vast number of antimicrobial agents. Phage therapy has again become a field worth of attention after years of abandonment in the western world. Phage application to humans and to animals is already being performed, nevertheless there is hardly any literature on studies concerning industrial environments and the use of phages as sanitation agents. Although the spoilage ability of *P. fluorescens *is well known, there are no studies involving the use of phages capable of infecting dairy industry isolates of this host species. Essentially, the work on Pseudomonad phages is concentrated on *P. aeruginosa *phages due to the clinical relevance of this strain, which is an opportunistic pathogen affecting mainly immunocompromised people and those suffering from cystic fibroses. There is some work done with *P. fluorescens *and phages focused on co-evolution studies [6-8]. So far, bacteriophages for *P. fluorescens *were never closer characterized in view of their physico-chemical, morphological, and life cycle properties.

Here we describe the isolation of phages for the milk spoilage bacterium *P. fluorescens *and the further characterization of one phage with a very high lytic efficiency. The data of this work indicate that this effective phage belongs to the T7-group of bacteriophages and that it may be a good sanitizing agent for control of environments where *P. fluorescens *may provoke a quality risk.

## Results

### Isolation and host range characterization of promising lytic phages

The aim of this work was to isolate, select and characterize an effective lytic phage for a range of *P. fluorescens *strains. Therefore, initially several phages were isolated from raw sewage of a wastewater treatment plant that receives effluents from different dairy plants. Isolation was performed using the enrichment method with different test species of *P. fluorescens*, which had been earlier isolated from Portuguese and United States dairy plants. These isolates include different ribotypes and also strains with different enzymatic activities (see Table 1). Not all *P. fluorescens *are capable of producing degradative defects in processed milk. Therefore isolates from New York dairy industries which were able to produce extracellular enzymes that are problematic for milk products and cause their respective spoilage [3,9,10] were included in this study. All initial tests were performed with NY *P. fluorescens *isolates positive for protease, lipase, and lecithinase activity (Table 1), with exception of two of the strains, D3-197 and B1-020 which were able to produce only one type of enzyme, protease or lipase, respectively.

**Table 1:** Characteristics of dairy *Pseudomonas *spp. used

**Strain**	**Species identification**	**Origin**	**Ribotype**	**Prot/Le/Li**	**Source or reference**
28	*P. fluorescens*	RSMT	*	+/*/*	PT unpublished
33	*P. fluorescens*	RSMT	*	+/*/*	PT unpublished
33B	*P. fluorescens*	RSMT	*	+/*/*	PT unpublished
35	*P. fluorescens*	RSMT	*	+/*/*	PT unpublished
37	*P. fluorescens*	RSMT	*	+/*/*	PT unpublished
37B	*P. fluorescens*	RSMT	*	+/*/*	PT unpublished
7	*P. fluorescens*	Teat cup shell	*	*/*/*	PT unpublished
8	*P. fluorescens*	Raw milk	*	*/*/*	PT unpublished
D3-149^‡^	*P. fluorescens*	Raw milk	409-S-3	+/+/+	[3]
D3-197^‡^	*P. fluorescens*	Processed milk	422-S-2	+/-/-	[3]
D3-331^‡^	*P. fluorescens*	Floor	57-S-8	+/+/+	[3]
D3-175^‡^	*P. fluorescens*	Processed milk	408-S-8	+/+/+	[3]
D1-045^‡^	*P. fluorescens*	Processed milk	50-S-8	+/+/+	[10]
B1-020^‡^	*P. fluorescens*	Potato isolate	57-S-5	-/-/+	[10]
D1-027^‡^	*P. fulva*	Raw milk	53-S-5	-/-/-	[10]
D2-160^‡^	*P. putida*	Raw milk	112-S-7	-/-/-	[10]
B1-041^‡^	*P. putida*	Raw milk	50-S-7	-/-/-	[10]
D1-026^‡^	*P. putida*	Raw milk	94-S-6	-/-/-	[10]
D1-046^‡^	*P. fragi*	Raw milk	72-S-3	-/-/-	[10]
B1-020^‡^	*P. fragi*	Raw milk	50-S-6	-/-/-	[10]

Isolate characteristics include origin, ribotypes and extracellular enzyme production [Protease (Prot), Lecithinase (Le) and Lipase (Li)]

RSMT – rubber short milk tube that connects the teatcup assemblies to the claw. * not performed, + positive for enzyme activity, - negative for enzyme activity.

^‡ ^kindly provided by K. Boor (Milk Quality Improvement Program, Department of Food Science, Cornell University, Ithaka, N.Y.). All other bacterial strains were isolated from the dairy industry plant in Pacos de Ferreira, Portugal.

Altogether 17 phages were isolated and showed lytic activity against some of the different bacterial strains (Table 2). All the isolated phages were used in further screening assays using the phage spot test on bacterial lawns of each different strains in order is to characterize their host range and to select the phage capable of infecting the greatest number of *P. fluorescens *strains. This screening procedure is the basis of phage typing methods and it enabled the differentiation of 14 different *P. fluorescens *strains as they resulted in dissimilar phage screenings profiles.

**Table 2:** Sensitivity of phages to different *P. fluorescens *isolates from dairy industry

Isolate	Phage ϕIBB-																
	PF7A	PF7B	PF7C	PF7D	PF8	PF37B	PF33A	PF33B	PF33C	PF33D	D3-197A	D3-197B	D3-149A	D3-149B	D3-331A	D3-331B	D3-331C

7	**C**	**C**	**C**	**C**	**C**	**C**	-	-	-	-	**C**	**C**	**T**	**T**	-	-	-
8	**C**	**C**	**C**	**C**	**C**	**C**	**C**	**-**	**-**	**C**	**C**	**C**	**C**	**C**	-	-	-
28	**C**	**C**	**C**	**C**	**C**	**C**	**C**	**-**	**-**	**-**	**T**	**T**	**C**	**C**	-	-	-
33	**C**	**C**	**C**	**C**	**C**	**-**	**C**	**T**	**T**	**T**	**T**	-	-	**C**	**T**	-	-
33B	**C**	**C**	**C**	**C**	**C**	**C**	**C**	**-**	**-**	**-**	**T**	**T**	-	-	-	-	-
35	**C**	**C**	**C**	**C**	**C**	**C**	**C**	**-**	**-**	**-**	-	-	**C**	**-**	-	**T**	**T**
37	**T**	**T**	**T**	**T**	**TT**	-	-	-	-	-	-	-	-	-	-	-	-
37B	**C**	**C**	**C**	**C**	**C**	-	-	-	-	-	**T**	-	-	-	-	-	-
D3-149	**C**	**C**	**C**	**C**	**C**	**C**	-	-	-	-	**C**	**C**	**C**	**C**	-	-	-
D3-197	**T**	**T**	**T**	**T**	**TT**	**C**	-	-	-	-	**C**	**C**	-	-	-	-	-
D3-331	-	-	-	-	-	-	-	-	-	-	-	-	-	-	**C**	**C**	**C**
D3-175	**C**	*	*	*	*	*	*	*	*	*	*	*	*	*	*	*	*
B1-020	**C**	*	*	*	*	*	*	*	*	*	*	*	*	*	*	*	*
D1-045	**C**	*	*	*	*	*	*	*	*	*	*	*	*	*	*	*	*
D1-027	**-**	*	*	*	*	*	*	*	*	*	*	*	*	*	*	*	*
D2-160	**-**	*	*	*	*	*	*	*	*	*	*	*	*	*	*	*	*
B1-041	**-**	*	*	*	*	*	*	*	*	*	*	*	*	*	*	*	*
D2-026	**-**	*	*	*	*	*	*	*	*	*	*	*	*	*	*	*	*
D1-046	**-**	*	*	*	*	*	*	*	*	*	*	*	*	*	*	*	*
B1-020	**-**	*	*	*	*	*	*	*	*	*	*	*	*	*	*	*	*

**Group**	**1**					**2**	**3**	**4**		**5**	**6**	**7**	**8**	**9**	**10**	**11**	

* not tested, C – clear phage plaque, T – turbid phage plaque, TT – very turbid, – no phage plaque

According to the phage lytic profiles, the phages were divided into 11 different groups based on the host susceptibility, as indicated on the bottom of Table 2. Each group is characterized by the same lytic profile of the phages for the same host strains, which either were sensitive to the distinct phages, by showing clear or turbid plaques, or resistant (no phage plaques observed). From these results it seems very likely that the phages belonging to different groups also represent different phages. Several phages were able to lyse most of the *P. fluorescens *isolates from the Portuguese dairy plant, however some phages were more host-specific, such as the phages isolated for *P. fluorescens *strains D3-331 and 33. Phages ϕIBB-D3-331A to C only formed clear plaques on the *P. fluorescens *isolate D3-331 and a turbid plaque in one more isolate. Phages ϕIBB-33B to D caused turbid plaques on the host which was used for isolation and only ϕIBB-33D was additionally able to produce clear plaques on host strain 8. The phages with the widest host range belonged to group 1 and where isolated with the *P. fluorescens *strains number 7 and 8 respectively. From the phage group 1, phage ϕIBB-PF7A had the largest plaque diameter (data not shown). Therefore, phage ϕIBB-PF7A was selected for further characterization studies and tested for lysis ability in other characterized *P. fluorescens *isolates and other *Pseudomonas *species provided (*P. putida*, *P. fragi *and *P. fulva*) by the Cornell University (NY) which were unable to produce extracellular enzymes. Phage ϕIBB-PF7A was only able to form plaques on *P. fluorescens *strains and did not lyse the three *Pseudomonas *species tested (Table 1).

*P. fluorescens *ribotypes tested where: 409-S-3, 422-S-2, 408-S-8, 50-S-8, and 57-S-5. Ribotypes 50-S-8 and 57-S-5 belong to *P. fluorescens *biovar II and the respective isolates, B1-020 and D1-045, cluster between the *P. fluorescens *and *P. putida *lineages in the so-called B3 cluster. Ribotypes 409-S-3, 408-S-8 and 422-S-2 have not been classified according to the biovar to which they belong. According to Dogan and Boor [3], from 42 *Pseudomonas *spp. isolated from four New Yok dairy plants, 21 isolates had *P. fluorescens *ribotypes. Furthermore, only 19% of the total *Pseudomonas *spp., or approximately 8 *P. fluorescens *ribotypes, are found in more than one dairy plant, while the other 81% are dairy plant specific ribotypes. Taking this into account and the fact that phage ϕIBB-PF7A could infect 13 different strains of which 5 were had different ribotypes suggests that ϕIBB-PF7A may be an interesting candidate for sanitizing applications and therefore was further characterized.

### Morphology of the phage particles

**Figure 1:**
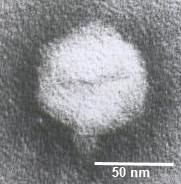
**Transmission electron micrographs of *P. fluorescens *phage ϕIBB-PF7A.**

Morphological characterization of phages using Transmission Electron Microscopy (TEM), one of the most used methods to classify phages, showed that phage ϕIBB-PF7A has a pentagonal outline indicating an icosahedral nature (Fig. 1). According to the TEM micrograph, phage ϕIBB-PF7A belongs to the *Podoviridae *family, which is characterized by phages with a short non-contractile tail. Phage ϕIBB-PF7A has a very short tail most likely belonging to type C in Bradley's classification [11]. Furthermore, the diameters of phage ϕIBB-PF7A's icosahedral capsid and tail length (Table 3) are similar to typical morphological values observed in members of the T7 phage group (H.-W. Ackermann, personal communication).

**Table 3:** Phage ϕIBB-PF7A plaque features, life cycle parameters and morphological characteristics determined from TEM micrographs

**Plaque**	Morphology*	**Life Cycle**
Diameter: 4–6 mm	Head size: 63 nm	Latent period: 15 min
Halo size: 0.5 mm	Tail size: 13 nm × 8 nm, tapering	Adsorption rate: 5.58 × 10^-10 ^mlmin^-1^
		Eclipse period: 10 min
		Rise period: 25 min
		Burst size: 153 PFU per infected cell

* Determinations performed by Dr. H. W. Ackermann, Laval University, Quebec, Canada.

### Phage DNA studies

**Figure 2:**
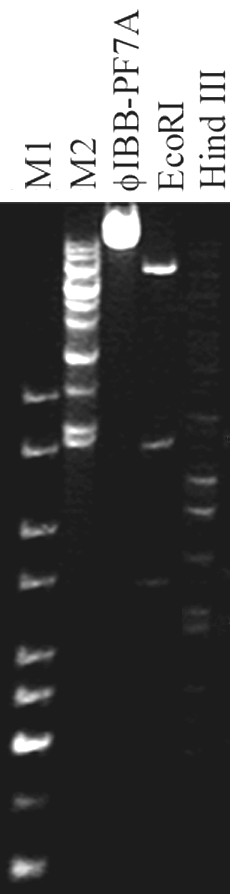
**Restriction analysis of phage ϕIBB-PF7A DNA.** Undigested phage DNA and phage DNA digested with EcoRI and HindIII. Lanes M1 and M2: 1 kb and lambda mix marker DNA ladders.

The isolation of phage DNA and the application of restriction enzymes allow the approximate determination of the genomic size of phages and evaluate if the size is consistent with the T7 classification proposed through the TEM observation. The restriction of phage ϕIBB-PF7A DNA was performed with EcoRI and HindIII. The sum of the fragments resulted in the following genomic sizes: 41,945 bp and 41,870 bp with EcoRI and HindIII, respectively (Fig. 2). The approximate genomic size of ϕIBB-PF7A with EcoRI and HindIII is approximately 42 kbp which is in the range of T7 phages that varies between 38,208 bp and 45,874 bp as further discussed below.

### Analysis of phage structural proteins

To further characterize ϕIBB-PF7A, its protein composition was analyzed by SDS-PAGE and 3 bands were N-terminally sequenced. SDS-PAGE allows the observation of the number of structural proteins present on this phage and to evaluate the similarities with protein profiles of known T7 structural proteins.

**Figure 3:**
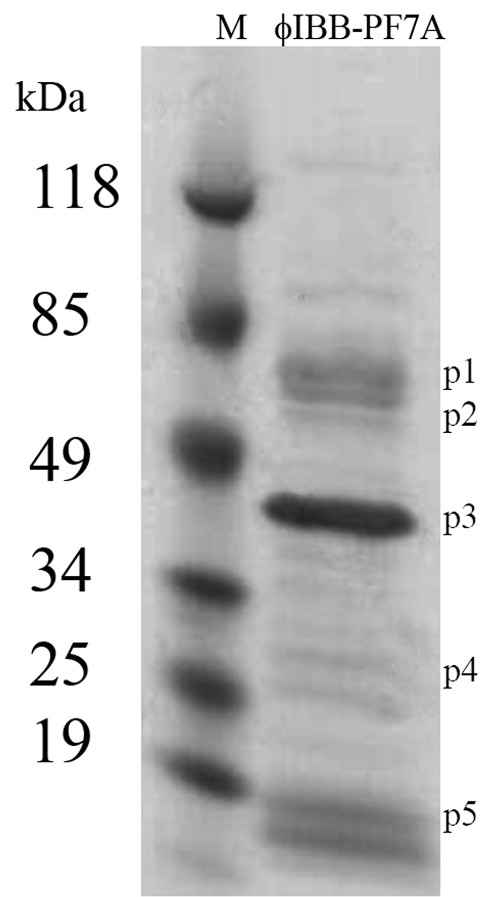
**SDS-polyacrylamide gel electrophoresis analysis of phage ϕIBB-PF7A structural proteins.** Phage lysate was mixed with Laemmli buffer containing SDS, boiled for 10 min, and loaded on a 4–20% gradient gel that was electrophoresed with Tris-glycine running buffer. Lane M: molecular weight marker. p1 to p5 mark sizes of typical T7 phage structural proteins. Further explanations in the Results section.

At least 16 bands can be clearly distinguished in the gradient gel (Fig. 3 and Table 4) ranging from approximately 16 to 140 kDa. The most predominant polypeptide band appeared at a size of approximately 45 kDa. This band (p3) could be assigned to the T7 major capsid protein 10A by its size and by its N-terminal sequence determination of the first 10 amino acid residues. This protein has 80% of sequence homology with ϕYeO3-12 and T3 phages, which are both members of the T7 phage supergroup. Also four other protein bands could be correlated with T7 structural proteins: T7 tail fiber protein in the monomeric form (p1 in Fig. 3); head-tail connector protein (p2), capsid assembly protein (p4), and also the internal virion protein B (p5) [12,13]. One of these four bands, the 63.1 kDa band (p1), gave a signal in the N-terminal sequence determination. The 63.1 kDa band resulted in 8 clearly determined amino acid residues and was 80% similar to a hypothetical protein of Thalassomonas phage BA3. Like ϕIBB-PF7A, phage BA3 also belongs to the *Podoviridae *family of phages but has not yet been further classified. The N-terminal sequence of the 50.5 kDa protein band did not give sequence which indicates that this protein is N terminally blocked. The analysis of ϕIBB-PF7A structural proteins clearly demonstrate that there are comparable proteins to other T7-like phages and like other phages belonging to this group, the most predominant structural protein is the major capsid protein 10A as verified by N-terminal sequencing.

**Table 4:** Phage ϕIBB-PF7A structural proteins

MW (kDa)^a^	Probable T7 protein	aa sequence	Identity (%)	BLASTp similarity (phage and accession nr.)	Alignment
137.3					

98.4					

85.6					

74.5					

68.5					

63.1 (p1)	Tail fiber (monomeric form of gp17)^1^	KEVLFGDS	80	Hyp. protein (phage BA3, YP001552271)	KEVLFG – DS KEVLFG – DS KEVLFGLNDS

59.7 (p2)	Head-tail connector (gp8)^1^				

50.5	-	No sequence	-	N-terminally blocked	-

45.2 (p3)	Major capsid (gp10A)^2^	AQMQGGQQIG	80	Major capsid protein 10A (phage ϕYeO3-12, NP_052109; phage T3, NP_523335)	AQMQGGQQIG A – QGGQQIG ANIQGGQQIG

39.3					

38.2					

30.6 (p4)	Capsid assembly (gp9)^1^				

29.0					

25.9					

18.6 (p5)	Internal virion B (gp14)^1^				

16.6					

Molecular weights of phage ϕIBB-PF7A structural proteins were determined from SDS-PAGE gels and compared with known T7 structural proteins with approximate weights, N-terminal sequence of proteins, similarities and respective accession numbers

^a) ^Average from 2 separate determinations; ^1 ^Kovalyova and Kropinski, 2003; ^2 ^Pajunen et al. 2000.

### Phage growth characteristics

**Figure 4:**
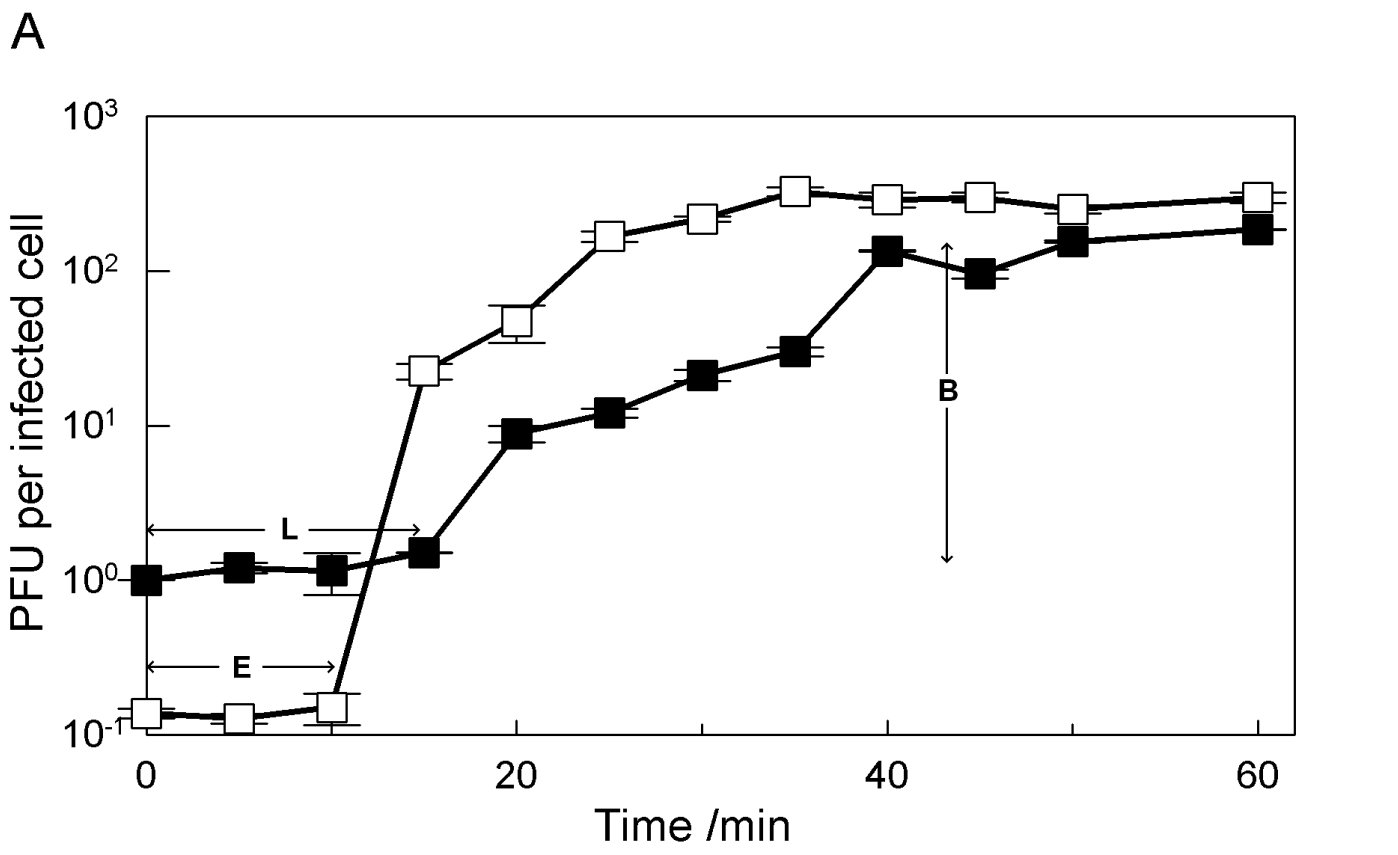
**One-step growth curve of phage ϕIBB-PF7A in *P. fluorescens *at RT.** Shown are the PFU per infected cell in untreated cultures (■) and in chloroform-treated cultures (□). The phage growth parameters are indicated in the figure and correspond to: E-eclipse period; L-latent period and B-burst size.

The EM, genomic and structural protein studies permit to classify ϕIBB-PF7A as a T7-like phage. Furthermore, it was our aim to characterize ϕIBB-PF7A's life cycle and adsorption ability. Firstly, one-step growth studies were performed to identify the different phases of a phage infection process. After infection of ϕIBB-PF7A's host, the *P. fluorescens *isolate nr. 7, the phage growth cycle parameters – the latent period, eclipse period, rise period, and burst size, were determined from the dynamical change of the number of free and total phages (Fig. 4 and Table 3). In the system studied, the eclipse and latent periods of ϕIBB-PF7A were very short, 10 and 15 min, respectively. ϕIBB-PF7A yielded a burst size of 153 PFU per infected cell after 25 min at RT. These phage life cycle values are in conformity with the values normally observed for T7 group phages.

**Figure 5:**
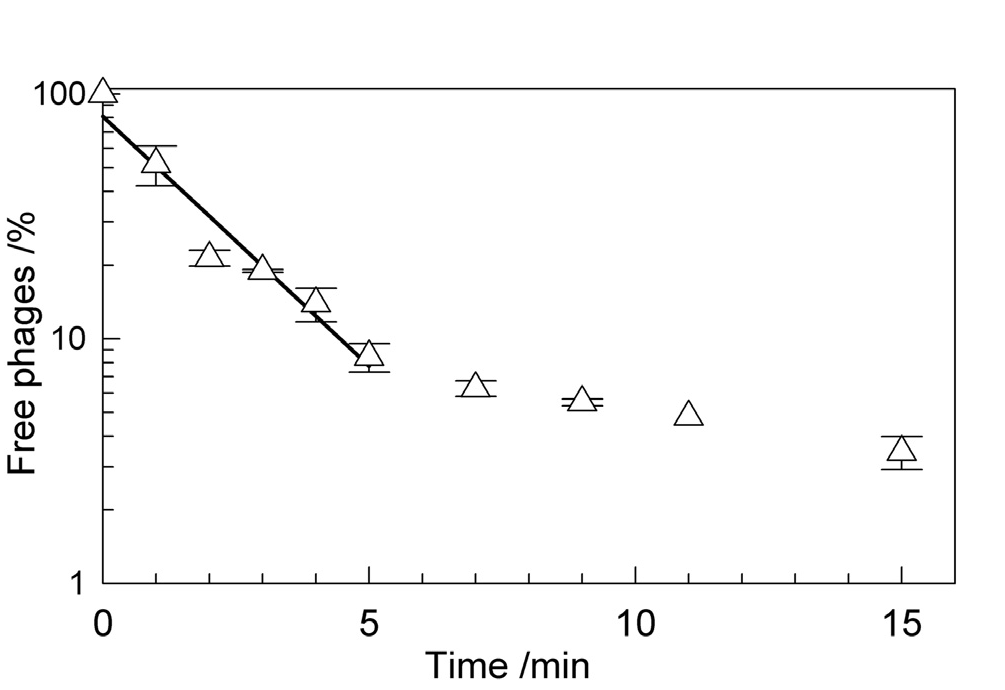
Percentage of free ϕIBB-PF7A phages after infection of steady-state *P. fluorescens *at a MOI of 0.01.

The adsorption efficiency of phages to the host was estimated with cells in the early logarithmic growth phase. The experiment was carried out at room temperature under constant shaking (150 rpm) and a phage inocculum of MOI = 0.01. The number of free phages was calculated from the PFU of chloroform-treated samples within 15 min after inoculation (Fig. 5). Phage ϕIBB-PF7A appears to have two adsorption phases: a very rapid adsorption to its host during the first 5 min is followed by a slower rate of attachment after 5 min. The number of free phages was below 10% already within 5 min and below 5% within 15 min after infection (Fig. 5). The adsorption rate, which represents the phage adsorption affinity towards the host, was calculated according to Barry and Walter [14] for a period of 5 min. The adsorption rate constant of phage ϕIBB-PF7A was calculated to be 5.58 × 10^-10 ^ml min^-1 ^(Table 3) which is similar to other T7 rates in literature [14].

## Discussion

*Pseudomonas fluorescens *is a major milk product contaminant as well as spoilage causing agent of fresh poultry and refrigerated foods, in particular of refrigerated meats [1,3,15-18]. Product contamination occurs at different stages of processing which shows that it is difficult to maintain the processing environment clean from this bacterium mainly due to the ecologic diversity and multiple origins of *Pseudomonas *spp. [3]. Phages have been suggested as alternative anti-microbial agents for a variety of pathogenic bacteria found in food processing environments [19-23]. So far, no phages have been closely studied for their ability to infect the spoilage causing bacterium *P. fluorescens*. The bacterial isolates tested in this work include different ribotypes and isolates capable of producing extracellular enzymes, such as protease, lecithinase and lipase that cause spoilage of milk products. A number of phages were isolated for *P. fluorescens*, some of which exhibited a high efficiency in lysing different *P. fluorescens *isolates while others had a narrower host range. This shows that a variety of lytic *P. fluorescens *phages can be easily isolated from the environment and their use as alternative sanitation agents will most likely be feasible for application in food and dairy industry as demonstrated by studies with *P. fluorescens *biofilms subjected to phage [24].

Of all the phages isolated, phage ϕIBB-PF7A was selected for further characterization. This novel highly lytic phage ϕIBB-PF7A has a strong virulence towards a number of *P. fluorescens *present on Portuguese and American dairy facilities. The verification of the ability of phage ϕIBB-PF7A in infecting isolates capable of producing extracellular enzymes isolates was an exceptionally important result and is an additional argument for phage based sanitation – it is possible to select phages only for a specific range of bacteria, in this case for the enzyme producing bacteria that are the major spoilage causative microorganisms.

The structural proteins are similar with known T7 proteins. Five distinct protein bands were identified according to their size representing the T7 tail fiber protein, the minor capsid protein, the major capsid protein 10A, the capsid assembly protein, and the internal virion protein B [12,13]. In fact, N-terminal sequencing identified one of the protein bands as the T7-like major capsid protein 10A.

Phage ϕIBB-PF7A has a genome size of about 42 kbp (Fig. 5) which is very close to the T7 phage ϕKMV (42,519 bp), a *Pseudomonas aeruginosa *phage [25]. This is well in the range of genome sizes of other T7-like phages; the smallest T7-like phage genome sequenced so far is the *E. coli *phage T3 having a size of 38,208 bp and the largest T7-like phage, a *Vibrio parahaemolyticus *phage, has a size of 45,874 bp [25].

The adsorption rate of 5.58 × 10^-10 ^ml min^-1 ^obtained through the one-step growth curve (Fig. 2) is also well in accordance with other T7-like phages, with values varying between 4.5 × 10^-10 ^and 8.9 × 10^-10 ^ml min^-1^, depending on whether the bacteria are alive or were killed [14].

The use of phages of the *Podoviridae *group, specifically T7-like phages, as therapeutic agents has been reported for different bacteria. *P. aeruginosa *T7-like phages ϕKMV, LKD16, and LKA1 have been suggested as good biological agents due to their ability to infect a variety of clinical *P. aeruginosa *isolates [25,26]. Phage PPpW-4 for *P. plecoglossicida *has been suggested equally suggested for combating the hemorrhagic ascites disease in cultured ayu fish [27,28]. Aside from the high rate of development of T7-like phages if e.g. compared to T4 phages that are being used in animal therapy, T7-like phages are also interesting for their small genome, which decreases the chance of transferring extended fragments of chromosomal DNA with pathogenicity islands [29] to host strains.

The data obtained in this study support the hypothesis that phage ϕIBB-PF7A can be a good candidate for phage based sanitation in food processing environments, preventing product spoilage due to extracellular enzyme producing *P. fluorescens *strains.

## Conclusion

To our knowledge, this is the first report with a detailed study of phages isolated for dairy *P. fluorescens *isolates. The newly isolated T7-like phage has an increased potential for lysing a variety of isolates and has several attractive features such as very short replication times and very fast adsorption ability which makes the phage a promising candidate for therapeutic or sanitation purposes.

## Methods

### Bacteria and growth conditions

The bacterial strains (see Table 1) were previously isolated from Portuguese and United States dairy plants. All bacteria were grown at 30°C in Tryptic Soy Broth (TSB, Fluka). Solid TSA medium contained 1.2% w/v of Bacto™ agar (Difco) and the soft agar top-layer contained 0.6% of Bacto™ agar. All bacteria were subcultured once and glycerol stocks were done and stored frozen at -80°C until further use.

### Bacteriophage isolation

Bacteriophages were isolated from a sewage treatment plant (ETAR de Esposende, Portugal). This wastewater treatment plant was selected because it receives effluents from many dairy industries and cow farms. For selection of phages 200 ml of raw sewage sample was added to a 1 L Erlenmeyer flask containing 200 ml of 2 × TSB medium and 50 μl of the respective bacterial test species with an optical density of 1.0. The solution was incubated for 24 h at 150 rpm and 30°C on a rotary shaker and was afterwards centrifuged (10 min, 10,000 × g, 4°C). The clear supernatant was chloroform treated and phage detection was done by spotting the phage lysate on bacterial lawns. These plates were incubated at 30°C for 12 h and inspected for plaques. Plaques were tested for containing only one single type of phage by repeated transfer of the lysates to new plates. Final lysates were stored at 4°C for further use.

### Host range of phages and selection of the most efficient phage

The isolated phages were investigated for host range specificity and lysis efficiency (no lysis, clear plaque, and turbid plaque) in screening tests against different *P. fluorescens*. Bacterial lawns of different *P. fluorescens *were propagated on TSA plates and 10 μl droplets of phages (1 × 10^4 ^up to 1 × 10^7 ^PFU ml^-1^) were put on the lawns. The plates were incubated 18 h and checked for presence of plaque. The most efficient phage, the one which was able to lyse the greatest number of hosts, was selected for further studies. The selection criteria included the lysis profiles, plaque clarity and size. Phages were named according to the bacteria from which they were isolated [e.g. phage ϕIBB-PF7 stands for Institute for Biotechnology and Bioengineering (IBB), the host bacterium *Pseudomonas fluorescens *(PF) and sample number 7]. In case different plaque sizes were obtained using the same host bacterium for phage isolation, the plaques were further purified and considered different phages and a caps letter was then added at the end of the phage name, i.e. phage ϕIBB-PF7A.

### Bacteriophage propagation and concentration

Small-scale concentration of phages was performed by spreading phages on the top-agar layer containing the respective host bacterium by plaque picking and using sterile paper strips. Briefly, 10 μl of isolated phage lysate was added onto a TSA Petri dish containing a soft top-agar layer with bacteria and spread with the help of sterile paper strips. After incubation the lysate from a clear Petri dish was eluted with SM buffer (5.8 g l^-1 ^NaCl, 2 g l^-1 ^MgSO_4 _× 7 H_2_O, 50 ml 1 M Tris, pH 7.5) and further purified with chloroform and stored at 4°C. These solutions were then used for preparing concentrated phage solutions in larger scale using the plate lysis and elution method as described by Sambrook & Russel [30] with some modifications. Briefly, a top agar was prepared containing 1 ml of phage solution and 1 ml of a bacterial overnight culture in 30 ml of soft-agar. This agar was added to 250 ml T-flasks with a thin bottom layer of TSA. After solidification of the top agar layer the T-flasks were incubated at 30°C overnight. Afterwards, the flasks were eluted with SM buffer and the phage lysate was first concentrated with PEG 8000 and then purified with chloroform. Samples in SM buffer were stored at 4°C until further use.

### Phage titration

Bacteriophage titer was analysed as described by Adams [31]. Briefly, 100 μl of diluted phage solution, 100 μl of a bacterial overnight culture, and 3 ml of molten agar were mixed in a glass tube and poured into a TSA containing Petri dish. Plates were incubated for 18 h after which plaque forming units (PFU) were counted.

### Electron microscopy

Bacteriophage particles were sedimented at 25,000 × g for 60 min using a Beckman (Palo Alto, CA) J2-21 centrifuge with a JA 18.1 fixed-angle rotor. Bacteriophages were washed twice in 0.1 M ammonium acetate (pH 7.0), deposited on copper grids provided with carbon-coated Formvar films, stained with 2% potassium phosphotungstate (PT, pH 7.2), and examined in a Philips EM 300 electron microscope (performed by Dr. H. W. Ackermann, Laval University, Quebec, Canada).

### SDS-PAGE

Purified phage solution was precipitated with 4 volumes of ice-cold acetone. After centrifugation (1,600 × g, 20 min, 4°C) the pellet was air-dried and resuspended in PBS buffer (8 g l^-1 ^NaCl, 0.2 g l^-1 ^KCl, 0.2 g l^-1 ^KH_2_PO_4_, 1.44 g l^-1 ^Na_2_HPO_4 _× 2H_2_O, pH 7.5). SDS-PAGE was carried out according to Laemmli [32]. Briefly, 24 μl of sample were added to 8 μl of 4 × Laemmli buffer and boiled for 10 min. Samples were then loaded to 4 – 20% PAGEr precast gels (Cambrex) and electrophoresed with Tris-glycine buffer. After electrophoresis the gels were stained with Bio-safe Coomassie (BioRad).

### DNA isolation and restriction enzyme digestion

Phage DNA was isolated using a Wizard Lambda Preps DNA purification system (Promega, Madison, Wis.) according to the manufacturer's instructions.

Phage DNA was digested with the following restriction enzymes: Eco RI, Eco RV, Hind III, and Tsp 509I according to the instructions provided by the manufacturer. All restriction enzyme digestions were performed in triplicate. Electrophoresis at 15 V for 24 h was performed with a 0.7% TAE agarose gel stained with ethidium bromide. A 1 kb DNA ladder and Lambda Mix Marker (both from Fermentas) were used as molecular weight markers to calculate the sizes of the phage DNA fragments.

### N-terminal amino acid sequencing of proteins

Phage proteins separated in 4 – 20% gradient gels were transferred to PVDF membranes (500V, 1.25 h) and stained with Bio-safe Coomassie brilliant blue solution (Bio-Rad) (1 min) and de-stained with 50% methanol. The membrane was rinsed with milliQ water for 5 min and let dry. The chosen bands were excised from the membrane and subjected to Edman chemistry for determining the N-terminal sequences. The sequencing was performed on a Procise™ 492 protein sequencer (PE Applied Biosystems).

### One-step growth curve

One-step growth curves were performed as described by Pajunen *et al. *[13] with some modifications. Briefly, 10 ml of a mid-exponential-phase culture were harvested by centrifugation (7,000 × g, 5 min, 4°C) and resuspended in 5 ml fresh TSB medium in order to obtain an OD_600 _of 1.0. To this suspension, 5 ml of phage solution were added in order to have a MOI of 0.001 and phages were allowed to adsorb for 5 min at room temperature. The mixture was than centrifuged as described above and the pellet was resuspended in 10 ml of fresh TSB medium. Two samples were taken every 5 min over a period of 1 h. The first sample was plated immediately without any treatment and the second set of samples was plated after treatment with 1% (vol/vol) chloroform to release intracellular phages.

### Bacteriophage adsorption

Bacteria in the steady-state growth phase were diluted in TSB to an optical density (OD_600_) of 1.0. Afterwards, 10 ml of the bacterial suspension and 10 ml of phage solution were mixed in order to give a multiplicity of infection (MOI) of 0.01. The mixture was incubated at room temperature with shaking (150 rpm, Rotamax 120, Heidolph) and samples were collected every minute during a total period of 15 min. Samples were immediately chloroform-treated, diluted and plated on TSA plates. After overnight incubation at 30°C phage plaques were counted.

## Authors' contributions

SS performed all practical experimental work and wrote the manuscript. PN contributed with his experiences in phage cultivation and JA with her competence in biofilm control. JA and PN supervised the work. The final manuscript was read and accepted by all coauthors.
